# Monitoring adverse drug reactions in the community settings

**DOI:** 10.2471/BLT.19.245019

**Published:** 2019-11-01

**Authors:** Ab Fatah Ab Rahman

**Affiliations:** a Faculty of Pharmacy, Universiti Sultan Zainal Abidin, Besut Campus, 22200 Besut, Terengganu, Malaysia.

Hospitals, clinics and pharmacies should abide by the five rights of medication use – the right drug to the right patient in the right dose by the right route at the right time. Yet in all stages of a medication process, that is, prescribing, transcribing, dispensing, administering and monitoring, medication errors and adverse drug effects still take place.

The prevalence of medication errors varies depending on the study design, definitions used and settings. For example, in Malaysian public hospitals and primary care clinics, the prevalence of medication errors has been reported to range from 11.7% (100/858)^1^ to 41.1% (720/1753).^2^ The reasons for medication errors also vary depending on setting. In contrast to hospital settings, administering and monitoring of medications in the community setting usually depend on the patients themselves or their family members, who are not always medication-literate. Both of these stages in medication-use processes are difficult to address, which increases the chances of introducing medication errors.

In Malaysia, reporting of medication errors and adverse drug events to the national reporting systems are voluntary.The reported number of medication errors has increased over the years, from 2626 reports in 2009 to 5770 reports in 2012.^3^ In 2012, an online system for reporting was launched, and in 2016 about 20 000 reports were received through this system.^4^ Hospital pharmacists reported most medication errors.^3^^,^^4^ Likewise, most reported incidences of adverse drug events were from hospital staff such as pharmacists, doctors and nurses. Health-care providers in the community setting such as private general practitioners and community pharmacists provided less than 5.0% (611/14 871) of reports of adverse drug events.^5^

The Malaysian online reporting system feeds into the national database, which shows that of the 19 923 reported medication errors most occurred in the prescribing (15 148) and dispensing (4089) stages, while the fewest errors occurred in the administering stage (251).^4^ Recently, a study of dispensing errors in six hospital pharmacies in Malaysia found 40.6% (76/187) labelling errors and 59.4% (111/187) filling errors.^6^ These findings should enable the government and policy-makers to develop intervention strategies to reduce or eliminate the occurrence of medication errors.

Reporting of adverse drug events to the Malaysian Adverse Drug Reaction Advisory Committee also showed an increasing trend: in 2018, the committee received 25 127 reports, an increase of approximately 350.0% compared to 2010 (5550 reports).^7^ Whether this trend reflects an actual increase in incidence of medication errors and adverse drug events or an increase in awareness to reporting among health-care providers in the country requires further investigation. 

Patients worldwide may also experience adverse drug events after they have been discharged from hospital; a recent study reported that the prevalence of medication errors after discharge from hospital to be around 51.3% (242/471).^8^ The occurrence of medication errors and adverse drug events among ambulatory patients in the community may be due to medication discrepancies or changes in medications that take place during transition of care. A medication discrepancy is the lack of agreement between different medication regimens during transition from acute care to post-acute care,^9^ while transition of care has been identified as one of the key action areas by the World Health Organization (WHO) Global Patient Safety Challenge on Medication Safety. 

Medication discrepancies place patients at risk of medication error and adverse drug events, as these patients have 31.0% higher chances of being readmitted within 90 days of hospital discharge.^9^ Recent data have shown that one third of visits to the emergency department of a teaching hospital in Malaysia were medication-related.^10^

Community pharmacists have an important role in monitoring medication use among patients in the community setting. In line with the recent *Jeddah Declaration on patient safety* *2019 *to promote medication safety in community pharmacies, pharmacists working in the community should be encouraged to share their knowledge and experience. Data from hospital settings facilitated the development and improvement in medication safety programmes, many of which were successful. Therefore, gathering information and identifying medication errors and adverse drug effects from the community setting is key to improving medication safety.
